# Smoking cessation after myocardial infarction: Findings from a cross-sectional survey in Armenia

**DOI:** 10.18332/tpc/174359

**Published:** 2023-12-11

**Authors:** Varduhi Hayrumyan, Arusyak Harutyunyan, Tsovinar Harutyunyan

**Affiliations:** 1 Turpanjian College of Health Sciences, American University of Armenia, Yerevan, Armenia

**Keywords:** smoking cessation, coronary heart disease, self-efficacy, quitting, myocardial infarction, tobacco-dependence

## Abstract

**INTRODUCTION:**

The effectiveness of smoking cessation in preventing myocardial infarction (MI) and reducing its recurrence, morbidity and mortality is well established. Only half of the patients quit or reduce smoking after hospitalization. The study examined smoking cessation practices and factors associated with it at 6–12 months after hospitalization among smoker patients diagnosed with MI.

**METHODS:**

A cross-sectional survey (2016–2017) was conducted among smoker adult patients who were diagnosed with MI and were hospitalized at the largest cardiac hospital (Nork-Marash Medical Center) in Armenia. Data collection was conducted via medical record review and an interviewer-administered telephone survey (n=230). The patients were classified as non-quitters or quitters (those had not smoked even a puff within the past 30 days). Multivariate logistic regression analysis was used to examine factors associated with smoking cessation at 6–12 months post-hospitalization addressing multicollinearity with two separate regression models.

**RESULTS:**

The mean age of participants was 58.3 years and 98.3% were males. Though almost all MI patients attempted to quit, only 52.2% were successful abstainers at 6–12 months after hospitalization. Significant predictors of quitting included higher self-efficacy (AOR=1.07; 95% CI: 1.03–1.11, p<0.001), lower tobacco dependence (AOR=0.81; 95% CI: 0.66–1.00, p=0.050), not having family members who smoked (Model 1: AOR=0.24; 95% CI: 0.08–0.70, p=0.009; and Model 2: AOR=0.24; 95% CI: 0.09–0.67, p=0.006), having other hospitalization after MI due to heart disease (Model 1: AOR=5.42; 95% CI: 1.50–19.65, p=0.010; and Model 2: AOR=4.20; 95% CI: 1.32–13.31, p=0.015), higher number of household members (Model 1: AOR=1.83; 95% CI: 1.27–2.64, p=0.001; and Model 2: AOR=1.68; 95% CI: 1.20–2.35, p=0.002), and having at least one comorbidity (Model 1: AOR=4.20; 95% CI: 1.47–12.04, p=0.008; and Model 2: AOR=3.74; 95% CI: 1.40–9.97; p=0.008).

**CONCLUSIONS:**

The study emphasized the need for integrating evidence-based cessation services and targeted help for hospitalized MI patients in Armenia. Interventions should aim to improve self-efficacy, effectively treat dependence, and consider patients’ social environment while providing cessation assistance.

## INTRODUCTION

Tobacco use is one of the most preventable causes of cardiovascular disease (CVD)^1^. It considerably increases the risks of CVD and coronary heart disease (CHD) mortality^1^. According to the Report of the Surgeon General (2014), smoking is accountable for about one in four CVD deaths in the US^1^. Different studies indicated a dose-response relationship between the risk of CHD and the number of cigarettes smoked per day^2^. The extensive evidence suggests that tobacco use at all levels increases the risk of developing CHD^1^. The greater risk was observed even among people who smoked <5 cigarettes per day^1^. A meta-analysis of 141 cohort studies reported that males and females who smoked even on average one cigarette per day had 48% and 57% higher risk of CHD compared to never smokers, respectively^3^. A recent systematic review, on the effect of smoking cessation interventions on secondary prevention of CVD, indicated reduced risk of secondary CVD among individuals who quit smoking in comparison to those who continue smoking after hospitalization, with a potential enhancement in overall quality of life attributed to smoking cessation^4^.

Despite the benefits of smoking cessation, only half of the patients quit or reduced smoking, and many patients continue or resume smoking after hospitalization due to MI^5^. Key determinants of successful smoking cessation of post-myocardial infarction (MI) patients include low dependence on tobacco^6,7^, higher self-efficacy^8^, longer hospital stay^9^, having no depression^10^, having better social support^11^, lower severity of MI^12^, getting smoking cessation advice from a physician^13^, and some sociodemographic characteristics (such as older age, higher education level, higher socio-economic status)^9^. The literature suggests that prior attempts to quit are also important for the success of quitting after MI^14^. Having a history of vascular disease^7^, prior cardiac events^6,7^, and other comorbidities such as chronic obstructive pulmonary disease^6^, were shown to be independent predictors of smoking resumption in several studies.

Tobacco use prevalence is remarkably high among the Armenian male population and is considered one of the highest in the European region. In 2022, daily smoking rate among men and women was 53.2% and 2.0%, respectively^15^. The health consequences of the high prevalence of smoking in Armenia are manifesting in higher rates of CHD morbidity and mortality. In 2015, CHD accounted for 29.1% of all deaths in Armenia; MI contributed to 8.4% of all deaths^15^. Although Armenia was the first among former Soviet countries to join the WHO Framework Convention on Tobacco Control (FCTC) in 2004, a study conducted in 2014 to measure the progress in the implementation of FCTC revealed that there was inadequate assistance available to smokers who were willing to quit^16^. In 2020, Armenia adopted a new national comprehensive tobacco control legislation harmonized with the FCTC; however, the implementation of the FCTC Article 14 remains unsatisfactory^17^. There is no tobacco dependence treatment training for healthcare professionals in their medical education curriculum. Moreover, a study conducted in Armenia in 2016 revealed that the tobacco dependence treatment guidelines are not well implemented in the medical practice and physicians are not using those guidelines as recommended^18^.

There has been no research conducted in Armenia to explore smoking cessation practices and the main factors associated with smoking cessation after experiencing MI. Such investigation could help to better organize the care and smoking cessation support for this group of patients, and improve the overall survival after MI in Armenia. The current study aimed to investigate the smoking cessation practices and the factors associated with smoking cessation at 6–12 months after hospitalization among smoker patients with a diagnosis of MI.

## METHODS

### Study design

The research team conducted a cross-sectional survey among adult patients who were hospitalized in the Nork-Marash Medical Center (NMMC) from March 2015 to August 2016 with a diagnosis of MI (MI patients afterward). The NMMC is one of the largest cardiac hospitals in Armenia. The medical record review was followed by a telephone survey to explore smoking cessation practices and associated factors.

### Data collection

**Figure 1:**
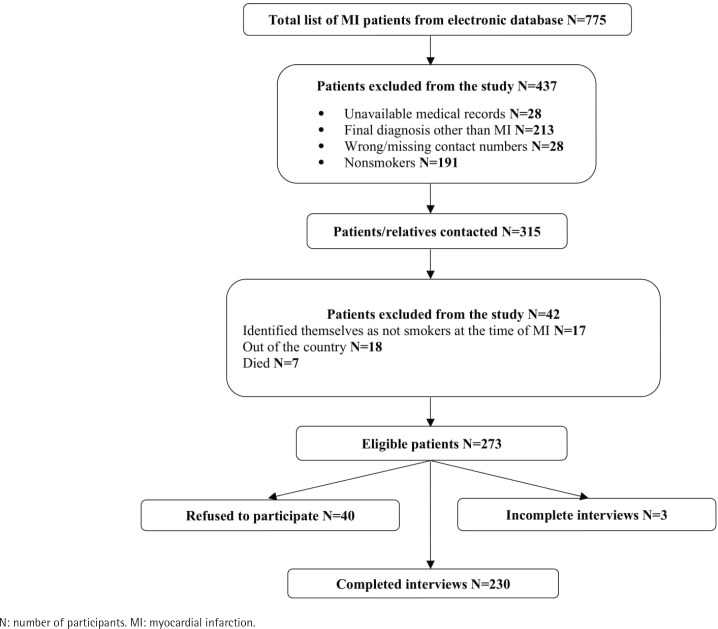
MI patients’ enrollment flowchart for a cross-sectional survey in Nork-Marash Medical Center, Armenia, 2016–2017 (N=230)

The data collection occurred in two phases, with the first phase conducted in March 2016 and the second in March 2017. This timeline was deliberately chosen to ensure that the patients interviewed were within the timeframe of 6–12 months after their hospitalization (as they were hospitalized from March 2015 to August 2016). After receiving permission from the NMMC, the hospital’s electronic database was used to obtain the list of MI patients hospitalized from March 2015 to August 2016 (n=775). Afterward, the medical records of those patients were reviewed to extract patient medical data by using the medical record abstraction form and identify eligible participants for the telephone survey. The names and contact information of patients were recorded in a separate journal form for interviewer-administered telephone interviews. Those patients who were aged ≥18 years, were smokers at the time of hospitalization, and had a final diagnosis of MI were eligible to participate in the telephone survey. As a result of the medical record review, about half of the patients (n=460) were excluded from the survey because of unavailable medical records (n=28), having a final diagnosis other than MI (n=213), having wrong or missing contact information in the medical records (n=28), and being non-smokers at the time of hospitalization (n=191). The patients whose smoking status was missing in their medical records (n=10) were also contacted by telephone. Overall, 315 patients were contacted for the telephone interview and 13.3% (n=42) of them were excluded from the study because of not meeting the inclusion criteria (17 patients identified themselves as non-smokers at the time of hospitalization), being outside of the country (n=18), or had died (n=7). Out of the remaining 273 eligible patients, 14.7% (n=40) refused to participate in the survey. As a result, 230 telephone interviews were completed (Figure 1). Oral informed consent was obtained from each participant before the telephone interview.

### Measures

The survey questionnaire was developed by the research team based on the existing literature and available survey instruments^19-21^. It was pre-tested (via telephone) among six MI patients before proceeding to data collection. The survey questionnaire consisted of several domains.

We examined the sociodemographic characteristics of study participants including age, gender, marital status, education level, employment, and average monthly income of the household. We also asked about the number of people living in the household and having a family member who smoked.

Regarding the smoking characteristics of participants^19^, we asked about the age of smoking the first cigarette, the age of starting daily smoking, and years of daily smoking. The level of nicotine dependence at the time of MI was measured by a brief 6-item Fagerström test for nicotine dependence (FTND). The items evaluate the amount of cigarette use, the compulsion to use tobacco, and nicotine dependence. The scale includes yes/no (scored from 0 to 1) and multiple choice (scored from 0 to 3) items. The total score can range from 0 to 10. The higher the FTND score the higher is the tobacco dependence^20^.

We assessed participants’ current smoking status by asking: ‘Have you smoked any cigarettes (even a puff) in the past 30 days?’ with response options ‘Daily’, ‘Less than daily’, and ‘Not at all’. The patients were classified as current smokers or quitters if they had not smoked even a puff within the past 30 days^5^. Current smokers were also asked about the number of cigarettes smoked per day.

We explored participants’ quit attempts by asking: ‘Have you ever made an attempt to stop smoking?’ with response options ‘No’ and ‘Yes’. We further explored whether they made quit attempts before or after the MI, the number of attempts made, and the longest duration of those attempts. We explored the reasons for the last quitting attempt (e.g. health concerns, advice from the physician, family pressure, advice and examples from others, abstinence during hospital stay, disliking smoking, severe health condition, cost, and realization of tobacco harms). We asked the participants about the methods of quitting during the last quit attempt (e.g. willpower; behavioral approaches such as staying away from smokers, distracting themselves, drinking tea, and others; medical measures like nicotine replacement therapy, cytisine, and varenicline; commercial cessation products; and other).

According to the clinical practice guidelines of tobacco dependence treatment, all tobacco users in clinical settings should receive brief intervention according to the evidence-based 5As approach^22^. The components of the approach include: ‘Asking’ patients about their smoking status, ‘Advising’ smokers to quit, ‘Assessing’ their willingness to quit (was not measured in this study), ‘Assisting’ patients to make a quit attempt, and ‘Arranging’ follow-up support during the quitting process^22^. We assessed the level of implementation with the ‘Ask’ component of the 5As model by checking whether the smoking status of patients was reported in their medical records. Then, during the survey, we assessed the self-reported level of implementation of the ‘Advice’, ‘Assist’ and ‘Arrange’ components. We asked the respondents to report if they have ever been advised by the physician to quit smoking after the MI. Also, we asked them to specify if they have received any assistance with quitting, including the provision of self-help materials, identification of a quit date, discussion of smoking cessation medications, prescription of smoking cessation medications, and whether the physician arranged follow-up support for smoking cessation after the MI.

Self-efficacy is confidence in personal ability to perform behaviors that bring desired outcomes^23^. We retrospectively measured self-efficacy at the time of MI by the Self-Efficacy Questionnaire (SEQ-12) ^21^. It is a widely used and validated tool consisting of 12 items that ask to indicate on a 5-point scale whether one could refrain from smoking in various high-risk situations from ‘1=Not sure at all’ to ‘5=Absolutely sure’. The SEQ-12 consists of two six-item subscales which respectively measure confidence in the ability to refrain from smoking when facing internal stimuli (e.g. feeling depressed) and external stimuli (e.g. being with a smoker). The sum of the 12 items of the scale yields a self-efficacy score ranging from 12 to 60 (higher score indicates higher self-efficacy)^21^.

The medical record abstraction form included information on the smoking status of patients, days of hospital stay; type of acute MI [non-ST segment elevation MI (NSTEMI) or ST-segment elevation MI (STEMI)], the number of diseased vessels (one diseased vessel was categorized as single vessel disease, while two and more diseased vessels were categorized as multiple vessel disease), and risk factors of CHD such as hypertension, body mass index (BMI) over 25 kg/m^2^, history of CHD, and hypercholesterolemia. We created a dichotomous variable ‘0=No, 1=Yes’ indicating whether the patient had any other risk factors for CHD. Additionally, we extracted information about patients’ comorbidities such as gastrointestinal (GI) diseases, diabetes, urogenital diseases, peripheral vascular disease (PVD), respiratory diseases, cerebrovascular disease, previous cardiac surgery, and others. For this variable, we also created a dichotomous variable ‘0=No, 1=Yes’ indicating whether the patient had at least one comorbidity. During the survey, we also asked about having other hospitalizations due to heart diseases after experiencing MI (‘0=No, 1=Yes’).

### Data analysis

The descriptive and bivariate analyses highlighted the characteristics of the study participants and compared the independent variables of interest between quitters and non-quitters. Following the descriptive analyses, we conducted multivariate logistic regression to explore associations between participants’ smoking status at 6–12 months after hospitalization and independent variables. All independent variables which were associated with smoking status at the time of the interview at p<0.1 level in bivariate analysis were tested for multicollinearity using Variance Inflation Factor statistics. There was a negative correlation between self-efficacy and FTND scores. Thus, two different multivariate regression models were developed: Model 1 with the self-efficacy score and Model 2 with the FTND score. Statistical significance for multivariable models was set at p<0.05. All analyses were conducted using SPSS 21 and STATA 13 software (SPSS Inc., Chicago, IL, USA, and Stata Corporation, College Station, Texas, USA).

## RESULTS

### Participants’ characteristics

According to the reviewed medical records, out of 534 patients, 86.0% (n=459) were males and 14.0% (n=75) were females. The smoking status was not recorded in 1.9% (n=10) of medical records. The point prevalence of smoking according to medical records of MI patients at the time of hospitalization was 63.5% (n=333), 71.3% and 16.2% among males and females, respectively.

Among 230 participants who completed the phone survey in 2016–2017, the vast majority were males (98.3%; n=226) (Table 1). The mean age was 58.3 years (SD=9.4). More than one-third of participants had higher education (38.5%; n=87) and more than half were unemployed (54.4%; n=123). The majority of participants were married at the time of the interview (88.8%; n=198). Many of the participants (40.1%; n=79) reported having a household monthly income of ≤100000 AMD (about 210 US$). Quitters had higher number of household members (4.5 vs 3.9, p=0.008). The majority of study participants (54.8%; n=126) had at least one smoker family member in their household, and quitters had fewer smoker household members (76.4% vs 35.0%, p<0.001) (Table 1).

**Table 1:** MI patients’ sociodemographic characteristics and bivariate comparisons of non-quitters and quitters at 6–12 months after hospitalization, findings from a cross-sectional survey in Nork-Marash Medical Center, Armenia, 2016–2017 (N=230)

*Characteristics*	*Total (N=230) (100%) n (%)*	*Non-quitters (N=110) (47.83%) n (%)*	*Quitters (N=120) (52.17%) n (%)*	p*
**Age** (years), mean (SD)	58.28 (9.38)	58.06 (10.25)	58.48 (8.55)	0.735
**Gender**				0.272
Male	226 (98.26)	107 (97.27)	119 (99.17)	
Female	4 (1.74)	3 (2.73)	1 (0.83)	
**Education level**				0.219
8 years or less/10 years	50 (22.12)	28 (25.69)	22 (18.80)	
Professional technical	89 (39.38)	45 (41.28)	44 (37.61)	
Institute/university/postgraduate	87 (38.50)	36 (33.03)	51 (43.59)	
**Marital status**				0.206
Single	9 (4.04)	7 (6.48)	2 (1.74)	
Married	198 (88.79)	92 (85.19)	106 (92.17)	
Widowed	11 (4.93)	7 (6.48)	4 (3.48)	
Divorced/separated	5 (2.24)	2 (1.85)	3 (2.61)	
**Employment status**				0.939
Employed	101 (44.69)	49 (44.95)	52 (44.44)	
Unemployed/student	125 (55.31)	60 (55.05)	65 (55.56)	
**Household monthly income** (AMD)				0.168
≤100000	79 (40.10)	39 (39.00)	40 (41.24)	
100001–200000	56 (28.43)	34 (34.00)	22 (22.68)	
>200000	62 (31.47)	27 (27.00)	35 (36.08)	
Number of people living in the household, mean (SD)	4.16 (1.72)	3.85 (1.64)	4.46 (1.74)	0.008
Having a smoker family member	126 (54.78)	84 (76.36)	42 (35.00)	<0.001

MI: myocardial infarction. AMD: 100000 Armenian Dram about 210 US$.

* p-values from t-tests and chi-squared tests examining differences between non-quitters and quitters. SD: standard deviation.

Per the medical record review (Table 2), the majority of patients (76.2%; n=163) had ST-segment elevated MI and more than half of patients (54.2%; n=123) had single-vessel disease. There was a significant difference in the number of diseased vessels between quitters and non-quitters (p=0.046). The mean duration of hospital stay was 4.8 days (SD=3.25). Most of the patients (58.0%; n=123) had a history of previous CHD, BMI >25 kg/m^2^ (63.0%; n=136), hypertension (72.6%; n=164), and at least one comorbidity (56.1%; n=129). Additionally, quitters were more likely to have hospitalizations after MI due to heart disease compared to non-quitters (32.5% vs 14.7%, p=0.002) (Table 2).

**Table 2:** MI patients’ medical characteristics and bivariate comparisons of non-quitters and quitters at 6–12 months after hospitalization, findings from a cross-sectional survey in Nork-Marash Medical Center, Armenia, 2016–2017 (N=230)

*Characteristics*	*Total (N=230) (100%) n (%)*	*Non-quitters (N=110) (47.83%) n (%)*	*Quitters (N=120) (52.17%) n (%)*	p*
**MI type**				0.577
STEMI	163 (76.17)	79 (74.53)	84 (77.78)	
NSTEMI	51 (23.83)	27 (25.47)	24 (22.22)	
**Severity of the disease**				0.046
Single vessel disease	123 (54.19)	57 (47.90)	66 (61.11)	
Multiple vessel disease	104 (45.81)	62 (52.10)	42 (38.89)	
**Days of hospital stay**, mean (SD)	4.83 (3.25)	4.52 (2.99)	5.11 (3.47)	0.193
**Risk factors for CHD at the time of hospitalization**				
Having at least one additional risk factor	212 (92.17)	100 (90.91)	112 (93.33)	0.494
Hypertension	164 (72.57)	80 (74.07)	84 (71.19)	0.627
BMI >25 (kg/m^2^)	136 (62.96)	40 (39.22)	40 (35.09)	0.531
History of CHD	123 (58.02)	54 (55.10)	69 (60.53)	0.425
Hypercholesterolemia	13 (6.05)	6 (5.94)	7 (6.14)	0.951
**Comorbidities at the time of hospitalization**				
Having at least one comorbidity	129 (56.09)	55 (50.00)	74 (61.67)	0.075
Gastrointestinal diseases	47 (20.52)	19 (17.43)	28 (23.33)	0.269
Diabetes	34 (14.85)	17 (15.60)	17 (14.17)	0.761
Urogenital diseases	34 (14.85)	14 (12.84)	20 (16.67)	0.417
Previous cardiac surgery	23 (10.04)	11 (10.00)	12 (10.08)	0.983
Peripheral vascular disease	24 (10.43)	11 (10.00)	13 (10.83)	0.836
Respiratory diseases	16 (6.99)	5 (4.59)	11 (9.17)	0.175
Cerebrovascular disease	7 (3.06)	4 (3.67)	3 (2.50)	0.608
Other	5 (2.17)	2 (1.82)	3 (2.50)	0.723
**Hospitalizations after MI due to heart disease**	54 (23.89)	16 (14.68)	38 (32.48)	0.002

MI: myocardial infarction. CHD: coronary heart disease. BMI: body mass index.

* p-values from t-tests and chi-squared tests examining differences between non-quitters and quitters. SD: standard deviation.

Out of 230 surveyed participants in 2016–2017, 52.2% quit smoking immediately after MI and 47.8% were smokers at the time of the interview (6–12 months after MI) (Table 3). The mean amount of daily smoked cigarettes at the time of the interview was 18.5 (SD=15.1). The mean score of FTND at the time of MI was 6.3 (SD=2.4) showing overall high tobacco dependence, and it was significantly higher in non-quitters compared to quitters (6.9 vs 5.7, p<0.001). The overall mean self-efficacy score was 30.6 (SD=15.7). Quitters reported higher self-efficacy compared to non-quitters (36.3 vs 24.3, p<0.001).

**Table 3:** MI patients’ characteristics according to the telephone survey and bivariate comparisons of non-quitters and quitters at 6–12 months after hospitalization, findings from a cross-sectional survey in Nork-Marash Medical Center, Armenia, 2016–2017 (N=230)

*Characteristics*	*Total (N=230) (100%) n (%)*	*Non-quitters (N=110) (47.83%) n (%)*	*Quitters (N=120) (52.17%) n (%)*	p*
**Smoking history**				
Age of the first cigarette, mean (SD)	17.00 (5.35)	16.92 (5.88)	17.07 (4.83)	0.833
Age of daily smoking, mean (SD)	19.53 (5.90)	19.34 (5.66)	19.71 (6.13)	0.634
Years of daily smoking, mean (SD)	35.11 (11.33)	35.39 (11.75)	34.86 (10.97)	0.723
FTND score, mean (SD)	6.25 (2.36)	6.85 (2.17)	5.71 (2.41)	<0.001
Self-efficacy score, mean (SD)	30.56 (15.68)	24.33 (10.92)	36.30 (17.19)	<0.001
Internal stimuli	14.66 (8.23)	11.43 (5.93)	17.61 (8.92)	<0.001
External stimuli	15.83 (8.32)	12.83 (6.23)	18.63 (9.05)	<0.001
**Current smoking characteristics**				
Smoking status, past 30 days				
Daily	98 (42.61)	98 (89.09)		
Less than daily	12 (5.22)	12 (10.91)		
Not at all	120 (52.17)		120 (100)	
Cigarettes smoked per day, mean (SD)		18.50 (15.07)		
**Smoking cessation**				
Ever made a quit attempt	219 (95.22)	99 (90.00)	120 (100)	
Time of quit attempts				0.124
Attempts only before MI	3 (1.37)	3 (3.03)	0 (0.00)	
Attempts only after MI	88 (40.18)	42 (42.42)	46 (38.33)	
Before and after MI	128 (58.45)	54 (54.55)	74 (61.67)	
**Quit attempts before MI**, mean (SD)				
Number of attempts	5.14 (8.55)	3.68 (3.60)	6.26 (10.83)	0.088
Longest duration of attempts (months)	8.13 (16.03)	8.11 (19.54)	8.15 (12.92)	0.989
**Quit attempts after MI**, mean (SD)				
Number of attempts	1.27 (1.14)	1.56 (1.59)	1.04 (0.46)	0.001
Longest duration of attempts (months)	6.15 (4.41)	2.04 (2.26)	9.44 (2.54)	<0.001
**Reasons for last quit attempt**				
Health concerns (for self and family members)	146 (67.91)	54 (56.25)	92 (77.31)	0.001
Advice from physician	126 (58.60)	58 (60.42)	68 (57.14)	0.628
Family pressure	35 (16.28)	19 (19.79)	16 (13.45)	0.210
Advice and examples from others	12 (5.58)	6 (6.25)	6 (5.04)	0.701
Started to dislike smoking	10 (4.65)	8 (8.33)	2 (1.68)	0.021
Abstinence during hospital stay	6 (2.79)	5 (5.21)	1 (0.84)	0.053
Severe health condition	6 (2.79)	2 (2.08)	4 (3.36)	0.572
Cost	4 (1.86)	0 (0.00)	4 (3.36)	0.130
Realized that smoking is harmful	4 (1.86)	3 (3.13)	1 (0.84)	0.218
Other	3 (1.40)	1 (1.04)	2 (1.68)	0.691
**Methods used for last quit attempt**				
Willpower	185 (86.45)	80 (84.21)	105 (88.24)	0.393
Behavior approach	22 (10.28)	10 (10.53)	12 (10.08)	0.916
Family help	13 (6.07)	4 (4.21)	9 (7.56)	0.308
Commercial cessation products	8 (3.74)	4 (4.21)	4 (3.36)	0.745
Medical measures	3 (1.40)	2 (2.11)	1 (0.84)	0.434
**5 As approach**				
Ask	226 (98.26)	109 (99.09)	117 (97.50)	0.357
Advice	211 (92.95)	103 (95.37)	108 (90.76)	0.175
Assist (provision of self-help materials)	1 (0.44)	1 (0.93)	0 (0.00)	0.476
Arrange	0 (0.00)	0 (0.00)	0 (0.00)	
**Barriers to quitting**				
Cravings for cigarette		55 (57.29)		
Loss of way to handle stress		42 (43.75)		
Influence of other smokers		23 (23.96)		
Fear of gaining weight		10 (10.42)		
Low self-control		22 (22.92)		
A lack of available cessation methods		2 (2.11)		
Other		8 (8.51)		
**Readiness to quit**				
Planning to quit in the next 6 months		21 (19.09)		
Planning to quit in the next 30 days		41 (37.27)		
Would like to cut down the amount of cigarettes		30 (27.27)		
Not planning to quit		18 (16.36)		
**Self-confidence in quitting attempt**				
Will be successful		23 (23.71)		
May be successful		25 (25.77)		
May succeed or fail		25 (25.77)		
Likely to fail		24 (24.74)		

MI: myocardial infarction. N: number of participants. FTND: Fagerström test for nicotine dependence.

* p-values from t-tests and chi-squared tests examining differences between non-quitters and quitters. SD: standard deviation.

The majority of participants (95.2%; n=219) reported having at least one quit attempt in their life and almost all of them made an attempt to quit after MI (98.6%; n=216). The mean number of quit attempts among the participants before MI was 5.1 (SD=8.6) with the mean longest duration of 8.1 months (SD=16.0). Almost all quitters quit smoking successfully after the first quit attempt after MI: the mean number of quit attempts after MI was 1.0 (SD=0.5). The mean longest duration of quit attempts among non-quitters who tried quitting after MI and relapsed was 2.0 months (SD=2.3). The main reported reason for their last quit attempt was the health concern for themselves and their family members (67.9%; n=146). Quitters were more likely to mention health concerns as a reason for quitting compared to non-quitters (77.3% vs 55.2%, p=0.001). Advice from a physician was the second most common reason (58.6%; n=126) for the last quitting attempt, followed by family pressure (16.3%; n=35). When asked about the method of the last quitting attempt, most participants mentioned willpower (86.5%; n=185). Only 1.4% (n=3) of participants used smoking cessation medications for quitting. The most common barriers to quitting were cravings for a cigarette (44.2%; n=95), loss of a way to handle stress (26.5%; n=57), and influence of other smokers (17.2%; n=37).

The majority of the medical records of surveyed participants contained information on smoking status (98.3%; n=226) (‘Ask’). The vast majority of participants (93.0%; n=211) reported receiving smoking cessation advice from their physicians (‘Advise’). Only one participant mentioned receiving self-help materials when asked about any form of smoking cessation assistance received from the physicians (‘Assist’). None of the participants mentioned arranging follow-up care for smoking cessation by their physicians (‘Arrange’).

### Factors associated with quitting at 6–12 months after hospitalization due to MI

Factors associated with quitting at 6–12 months after hospitalization due to MI in the multivariate regression analysis were higher self-efficacy score (AOR=1.07; 95% CI: 1.03–1.11, p<0.001), lower FTND score (AOR=0.81; 95% CI: 0.66–1.00, p=0.050), not having smoker family members (Model 1: AOR=0.24; 95% CI: 0.08–0.70, p=0.009; and Model 2: AOR=0.24; 95% CI: 0.09–0.67, p=0.006), having other hospitalization after MI due to heart disease (Model 1: AOR=5.42; 95% CI: 1.50–19.65, p=0.010; and Model 2: AOR=4.20; 95% CI: 1.32–13.31, p=0.015), a higher number of people living in the household (Model 1: AOR=1.83; 95% CI: 1.27–2.64, p=0.001; and Model 2: AOR=1.68; 95% CI: 1.20–2.35; p=0.002), and having at least one comorbidity (Model 1: AOR=4.20; 95% CI: 1.47–12.04, p=0.008; and Model 2: AOR=3.74; 95% CI: 1.40–9.97, p=0.008) (Table 4).

**Table 4:** Multivariate logistic regressiona examining correlates of quitting smoking of MI patients at 6–12 months after hospitalization, findings from a cross-sectional survey in Nork-Marash Medical Center, Armenia, 2016–2017 (N=230)

*Variables*	*Model 1*		*Model 2*	
	*AOR (95% CI)*	p*	*AOR (95% CI)*	p*
Self-efficacy score	1.07 (1.03–1.11)	<0.001		
FTND score			0.81 (0.66–1.00)	0.050
Having smoker family member (Ref. No)	0.24 (0.08–0.70)	0.009	0.24 (0.09–0.67)	0.006
Health concern (for self and family members) (Ref. No)	2.82 (0.96–8.26)	0.058	2.58 (0.93–7.11)	0.068
Other hospitalization after MI due to heart disease (Ref. No)	5.42 (1.50–19.65)	0.010	4.20 (1.32–13.31)	0.015
Number of people living in the household	1.83 (1.27–2.64)	0.001	1.68 (1.20–2.35)	0.002
Multiple vessel disease (Ref. single vessel disease)	1.47 (0.52–4.12)	0.467	1.76 (0.67–4.60)	0.251
Having at least one comorbidity (Ref. No)	4.20 (1.47–12.04)	0.008	3.74 (1.40–9.97)	0.008
Number of quit attempts before MI	1.13 (0.99–1.28)	0.069	1.11 (0.98–1.26)	0.093

MI: myocardial infarction. FTND: Fagerström test for nicotine dependence. aVariables associated with smoking status (p<0.1) in bivariate analysis were examined for multicollinearity using Variance Inflation Factor statistics. Due to a negative correlation between self-efficacy and FTND scores, two separate multivariate regression models were developed: Model 1 (self-efficacy score) and Model 2 (FTND score).

* Statistical significance for multivariable models was set at p<0.05.

## DISCUSSION

This was the first study conducted in Armenia to explore smoking cessation practices and the factors associated with smoking cessation at 6–12 months after hospitalization among smoker patients with a diagnosis of MI. The study showed that the prevalence of smoking among MI patients in our sample was substantially higher (71.3% among males and 16.2% among females) compared to the general adult population of Armenia (53.2% among males and 2.0% among females)^15^, most likely reflecting its proven detrimental effect on heart health. Although almost all participants (98.6%) attempted to quit smoking after MI, only about half of the MI patients (52.2%) successfully quit smoking and maintained abstinence at 6–12 months after hospitalization. This quitting rate closely resembles the rates reported in similar studies conducted worldwide^5^.

Diseases highly associated with smoking, such as CHD, greatly trigger smoking cessation attempts and patients become more willing to receive smoking cessation interventions^24^. Indeed, the most common reason for quitting smoking reported by our study participants was health concerns for themselves and their family members (67.4%). Likewise, health concern was the main motivator to quit smoking in other studies conducted to identify the motivators of and barriers to quitting smoking^25^. Moreover, having at least one comorbidity and other hospitalization due to heart health increased the likelihood of quitting at 6–12 months after MI in our study, suggesting that poor health condition largely determines abstinence. However, some studies have shown that having a health condition can be a good motivating factor but it is not enough for successful long-term quitting, and interventions are recommended^26^.

Almost all patients in our study quit smoking successfully after the first quit attempt after MI. However, many study participants relapsed within a short period of time, which is in accordance with study findings reported by Vogiatzis et al.^6^, where most relapses occurred during the first 3 months following the discharge. This can be explained by the fact that most of the surveyed participants (86.5%) attempted to quit smoking by using only their willpower without any assistance. Other studies also found that relying on their own will while quitting is very common among smokers^27^. Yet a very low percentage of smokers attain prolonged abstinence at 6–12 months after the unaided quit attempt^28^.

The reasons behind this high proportion of unaided quitting might be numerous. Patients might have a lack of knowledge about smoking cessation methods and their effectiveness^29^. Moreover, most of the physicians do not fully comply with the evidence-based recommended tobacco dependence treatment interventions due to insufficient knowledge and inadequate training^18^. Our study demonstrated limited implementation of the recommended standard smoking cessation care among MI patients by their healthcare providers. On the one hand, the study showed high compliance with the ‘Ask’ and ‘Advise’ components of the recommended 5As smoking cessation approach, on the other hand, the ‘Assist’ and ‘Arrange’ components were absent or not applied properly. Overall, these patterns are comparable to what was found in other studies showing that most of the physicians fail to comply with all of the components of recommended the 5As approach, and they mainly fail to adhere to the last ‘Assist’ and ‘Arrange’ components^30,31^. Another possible reason could be a lack of smoking cessation pharmacotherapy products available in Armenia^18^.

While smoking cessation advice received from the physicians was the second most common reason for the patients’ quit attempt, indicating the significance of physicians’ role in quitting smoking, it did not seem to predict abstinence at 6–12 months in this study. This shows that partial intervention seemed not to be sufficient and recommended strategies should be fully and intensively implemented to increase chances of successful abstinence^32^. A study in Switzerland demonstrated that an active approach providing comprehensive smoking cessation behavioral intervention for all individuals hospitalized for acute coronary syndrome significantly boosts the acceptance of smoking cessation counseling and has the potential to enhance rates of smoking abstinence at 12 months^32^. It is also possible, that because of MI patients’ high nicotine dependence level, successful quitting was even more challenging when compared to other smokers^31^. The study showed that the level of nicotine dependence was a negative predictor of quitting smoking at 6–12 months after hospitalization due to MI and was consistent with the available literature^6,7^.

In agreement with the literature, self-efficacy was another important predictor of quitting after MI^8^. Thus, patients who were more confident in their ability to refrain from smoking in various situations were more likely to be abstinent at 6–12 months after experiencing MI. Self-efficacy is known to influence various types of health-related behaviors, with its importance particularly pronounced in behaviors of progressive complexity or difficulty^23^. While it has been shown that perceived efficacy to resist the addictive behaviors in high-risk situations can influence self-control regardless of how dependent people had become on a particular substance^33^, its direct relationship with dependence has not been fully explained. The strong negative correlation of self-efficacy with nicotine dependence score found in our study possibly reflects reciprocal influences of these constructs and warrants more nuanced exploration in future investigations.

The study revealed that having at least one smoker family member was negatively associated with smoking cessation among post-MI patients at 6–12 months after hospitalization, which means that those who had a smoker family member had a higher tendency to continue smoking after hospitalization. Likewise, studies showed that having smoker family members or peers strongly influenced the long-term success of smoking cessation^34^. Moreover, smokers who live in a social environment with a higher number of smokers have higher exposure to smoking cues and more positive norms towards smoking, thus increasing the likelihood of unsuccessful quitting^35^. The current study also showed that those having more household members were more likely to be abstinent at 6–12 months after MI. This finding is also in line with the literature suggesting that compared to those living alone, living with others, especially with non-smoker family members, increases the likelihood of successful cessation^36^. The study builds upon the existing literature on the importance of social context for smoking cessation attempts and highlights the significance of household composition and smoking characteristics of family members in the cessation outcomes of MI patients.

### Limitations

This study has several limitations. First, the study relied upon self-reported smoking status of participants and did not use any biochemical validation method to verify it. Second, since some variables were collected retrospectively including the history of smoking and quit attempts, tobacco dependence level, and self-efficacy, a recall bias cannot be ruled out. Third, some factors that might have influenced quitting smoking among MI patients such as social support and depression were not included in the analysis^10^. Fourth, the participants were sampled from a single healthcare facility in Yerevan which may limit the generalizability of the results to other medical settings and populations. Sixth, the study was conducted several years ago. However, it is important to note that although some changes may have occurred since the completion of the study, the landscape of smoking cessation in Armenia has remained relatively stable and neither significant alterations in the smoking cessation patterns or initiatives during this period have been extensively reported nor observed. Thus, the impact of potential developments following the study remains relatively minimal, and the fundamental dynamics of smoking cessation are expected to have persisted in a comparable manner.

## CONCLUSIONS

Despite the benefits of quitting, many smoker MI patients continue smoking at 6–12 months after hospitalization. While the severity of the health condition plays a vital role in initiating quitting attempts, the importance of assisting hospitalized patients in cessation efforts should not be underestimated. The study highlighted the need for the integration of recommended standard smoking cessation services in medical care and targeted assistance for all MI patients. The assessment of the nicotine dependence level is crucial for healthcare providers to appropriately tailor smoking cessation interventions. Moreover, our findings stress the importance of behavioral interventions that could focus on improving self-efficacy to increase the chances of long-term abstinence. Additionally, healthcare providers might consider targeting family members and friends for better quitting outcomes in MI patients.

This study has important practical implications for Armenia and other low- and middle-income countries where the FCTC Article 14 implementation is not yet satisfactory. The examined factors could guide the development of appropriate strategies to integrate tobacco dependence treatment into the healthcare system and improve long-term quitting outcomes and overall survival after MI.

## Data Availability

The data are available from the corresponding author on reasonable request.
